# Supplementary material for: Transposable Element Populations Shed Light on the Evolutionary History of Wheat and the Complex Co‐Evolution of Autonomous and Non‐Autonomous Retrotransposons
Source: Adv Genet (Hoboken). 2021 Dec 9;3(1):2100022. doi: 10.1002/ggn2.202100022 (PMC9744471; doi:10.1002/ggn2.202100022)
Supplement: Supplementary file 1 — Supporting Information [file GGN2-3-2100022-s002.pdf]

Supplementary Table 1. Numbers of identified full-length *RLC_Angela* retrotransposons in 10 sequenced wheat lines.

Wheat line	Retrotransposons
Arina*LrFor*	24,521
Chinese Spring	19,859
Jagger	22,095
Julius	22,795
LongReach Lancer	20,958
CDC Landmark	19,944
Mace	24,225
Norin 61	23,672
Spelt	28,353
CDC Stanley	24,282
SY Mattis	24,181

Supplementary Table 2. Chromosomal introgressions form different wheat lines which were used for TE population analysis. Introgressions were used if they contained at least 40 copies of either the *RLC_Angela* β or δ sub-pupulation.

Introgression	Start[a]	End[a]	Size [Mb]	RLC_Angela sub-pop. β	RLC_Angela sub-pop. δ	Wheat lines
1A292	292	462	170	93	62	Norin 61
2A203	203	286	83	N.A.	39	Arina*LrFor*, SY Mattis
2A726	726	781	55	45	70	Norin 61
2A738	738	787	49	33	42	CDC Landmark
3A696	696	751	55	48	N.A.	Norin 61
6A467	467	534	67	47	30	LongReach Lancer, Norin 61
7A005	5	59	54	50	39	CDC Stanley
7A108	108	198	90	56	42	CDC Landmark
7A559	559	639	80	42	32	Mace
2B093	93	572	479	159	N.A.	LongReach Lancer
2B123	123	287	164	68	71	SY Mattis
2B363	363	631	268	91	77	CDC Stanley
2B682	682	796	114	66	35	Mace
5B508	508	610	102	64	47	Norin 61
6B143	143	264	121	69	39	Julius, SY Mattis
6B144	144	292	148	79	48	LongReach Lancer, Mace
6B532	532	633	101	63	35	Mace
7B542	542	669	127	N.A.	51	Norin 61
2D361	361	483	122	62	122	Mace
2D512	512	563	51	27	51	Mace
2D571	571	619	48	11	48	Arina*LrFor*, Jagger, Julius, SY Mattis
3D470	470	510	40	20	40	LongReach Lancer
5D249	249	315	66	46	66	Norin 61

[a]Start and end position of the introgression in Mb.

Supplementary Table 3. Unique, recent insertions of RLC_Angela retrotransposons that were found exclusively in one of the 10 wheat genomes and for which orthologous loci that do not contain the insertion could be identified in other wheat genomes.

Chromosome	Position	length [bp]	Orientation
Lancer_v1_chr7B	370024608-370033239	8631	forward
Stanley_v1_chr2B	322730137-322738803	8666	forward
Stanley_v1_chr5B	602215205-602223730	8525	forward
Stanley_v1_chr5B	602215205-602223730	8525	forward

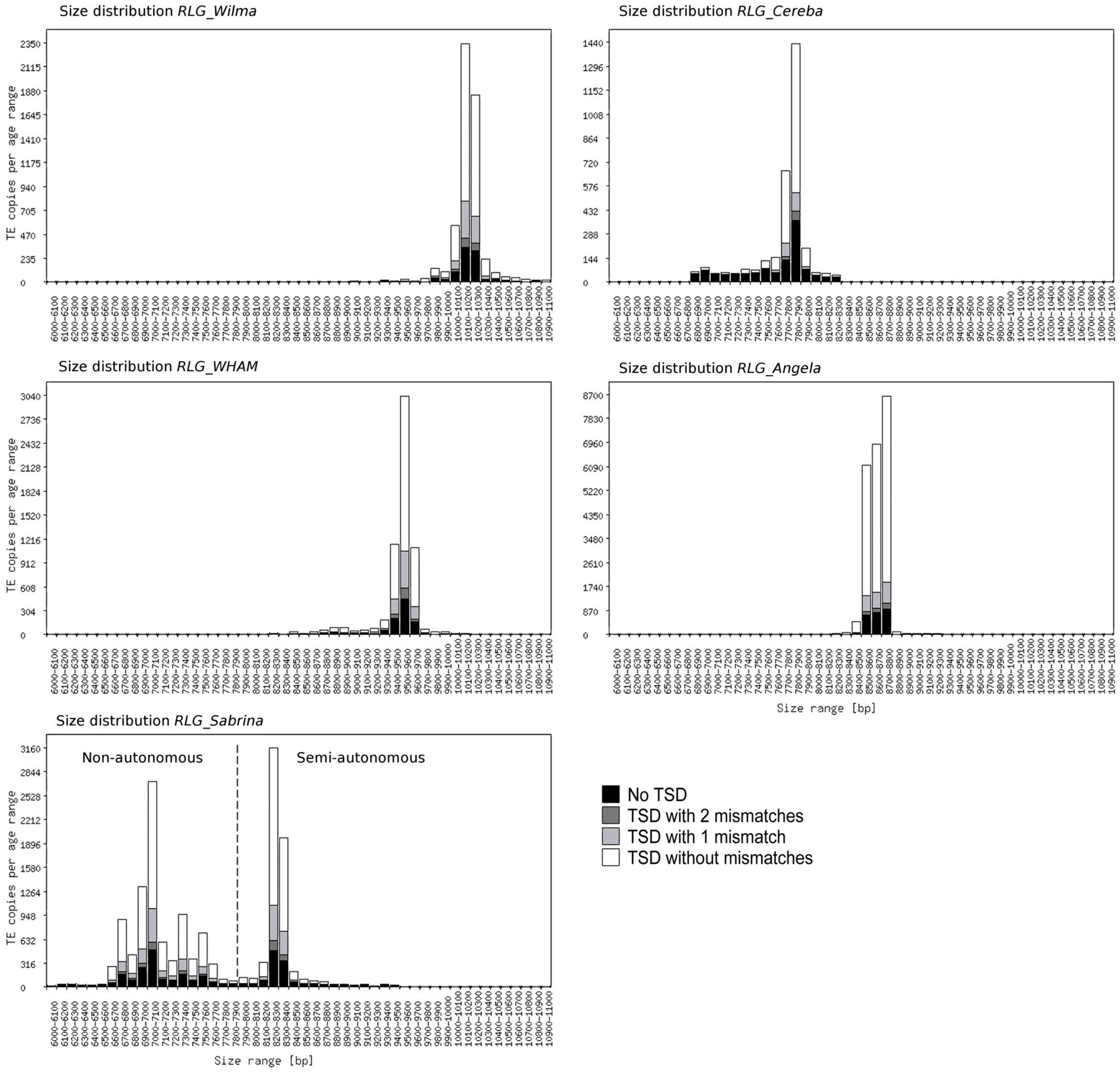

Supplementary Fig. 1. Size distribution of full-length LTR retrotransposons identified in the genome of bread wheat (*Triticum aestivum*). The x-axis shows the size range in bp, while the y-axis shows the number of full-length retrotransposon per respective size range. The number for retrotransposons with and without target site duplications (TSDs) are stacked on top of each other.

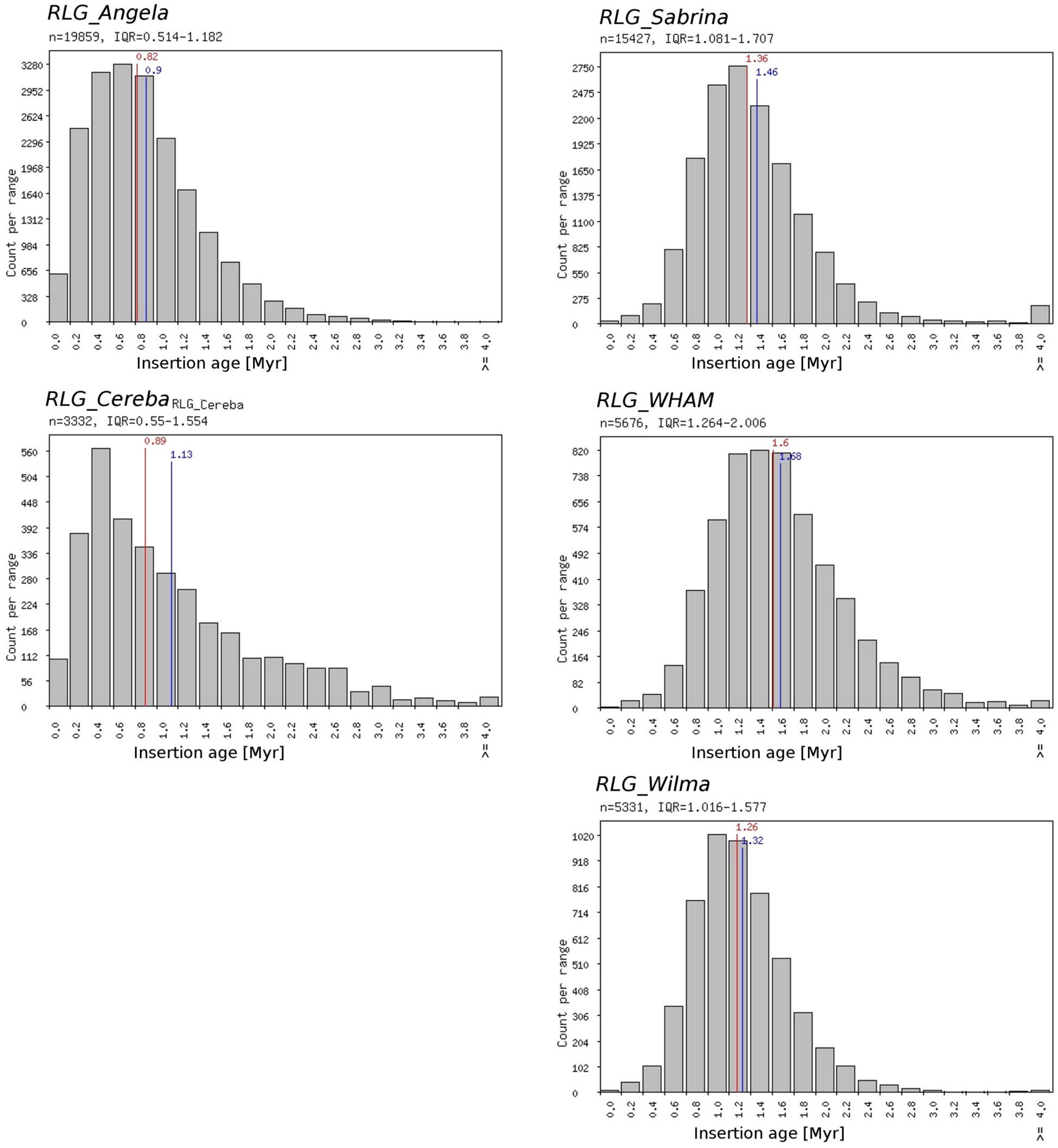

Supplementary Fig. 2. Insertion age distributions of full-length LTR retrotransposon families in wheat. The x-axis shows insertion age range in millions of years, while the y-axis shows the number of retrotransposons found in each range. (Myr: Million years, n: number of full-length elements, IQR: inter-quartile range, i.e. the range in which 50% of all values lie).

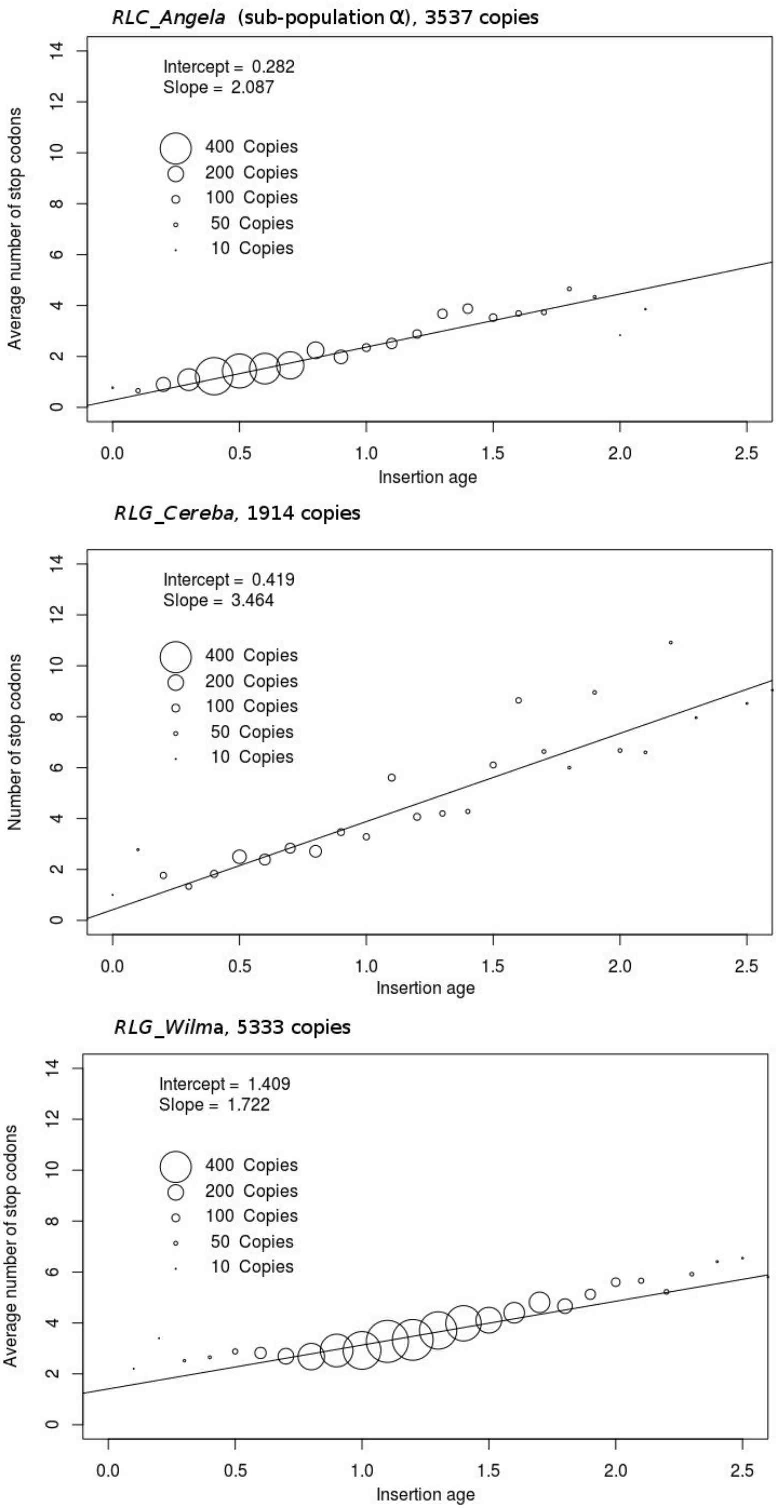

Supplementary Fig. 3. Numbers of in-frame stop codons in CDS of *RLC_Angela* (top), *RLG_Cereba* (middle) and *RLG_Wilma* (bottom) retrotransposons. The x-axis is the insertion age in million years (Myr), while the y-axis shows the average number of in-frame stop codons in the CDS for each insertion age group. TE copies were analyzed in insertion age bins of 100,000 years. The sizes of the circles indicate the number of TE copies in each bin. All age estimates and numbers of stop codons were used for linear regression. Where the line intercepts with the y-axis is the extrapolated average number of in-frame stop codons in the CDS of newly inserted TE copies. Note that the y-axis labels are shifted a bit to the left to allow the display of data points with x values of zero. Note that the slope of the regression line differs in the three TE families while the intercept values is very similar in all. The regression analysis indicates that newly inserted TE copies have on average roughly 0.2-0.4 in-frame stop codons (i.e. about 20-40% of all newly inserted copies have a defective ORF).

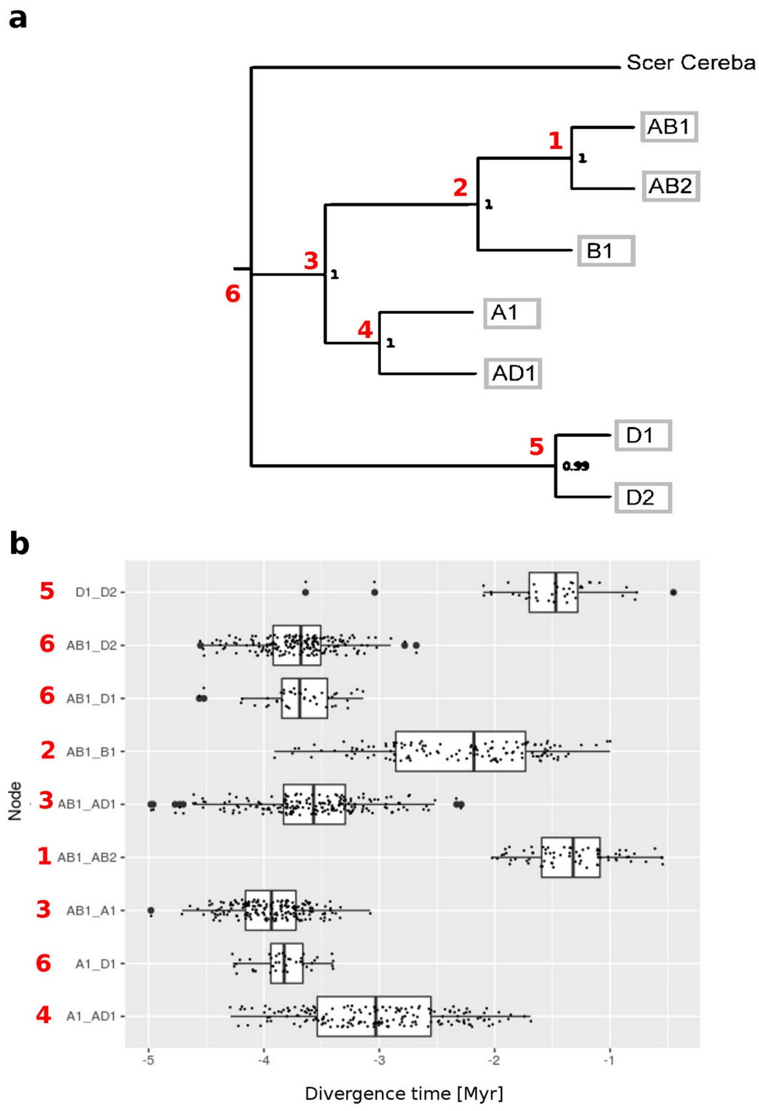

Supplementary Fig. 4. Pyhlogenetic analysis and divergence time estimates of *RLG_Cereba* subfamilies. **a.** Phylogenetic tree at the top derived from predicted polyproteins of consensus sequences for *RLG_Cereba* subfamilies. A rye *RLG_Cereba* protein was used as outgroup (Scer_Cereba). **b.** Individual nodes (indictaed in red) were dated based on numbers of nucleotide substitutions in fourfold degenerate CDS sites (see methods). Here, we randomly picked between 44 and 189 pairs of CDS from the respective subfamilies and aligned them to determine divergence time based on numbers of synonymous substitutions. The boxplots show the distribution of divergence time estimates for the individual CDS pairs. For nodes 3 and 6, multiple estimates were made by using pairs from different branches The x-axis is the divergence time in million years, while the y-axis separates the comparisons that were used to date the nodes.

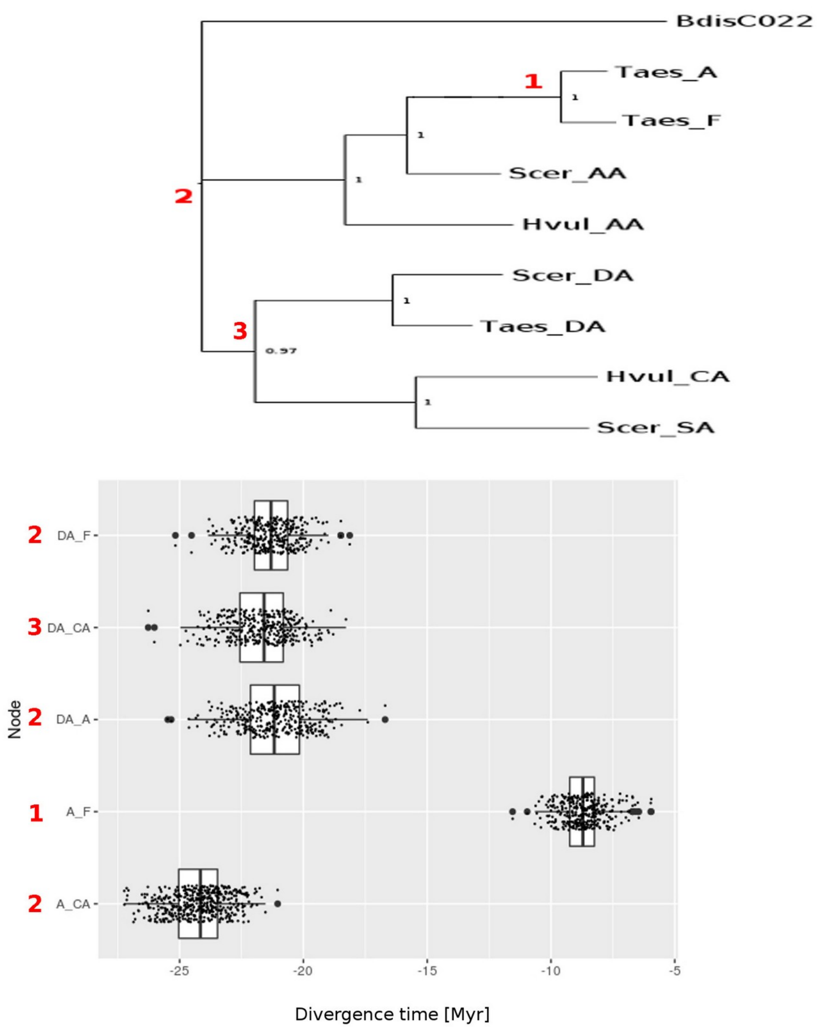

Supplementary Fig. 5. Pyhlogenetic analysis and divergence time estimates of *RLC_Angela* sub-populations. **a.** Phylogenetic tree derived from predicted polyproteins of *RLC_Angela* subfamily consensus sequences. Predicted protein sequences rye (prefix Scer), barley (prefix Hvul) and *Brachypodium distachyon* (prefix Bdis) were used as internal references and outgroup, respectively. **b.** Three nodes (indictaed in red) were dated based on numbers of nucleotide substitutions in fourfold degenerate CDS sites. Here, the predicted CDS of randomly picked TE pairs of sequences from the respective subfamilies were aligned. The histograms show the distribution of divergence time estimates for the individual TE CDS pairs. For node 2, multiple estimates were made by using pairs from different branches. The x-axis is the divergence time in million years, while the y-axis separates the comparisons that were used to date the nodes.

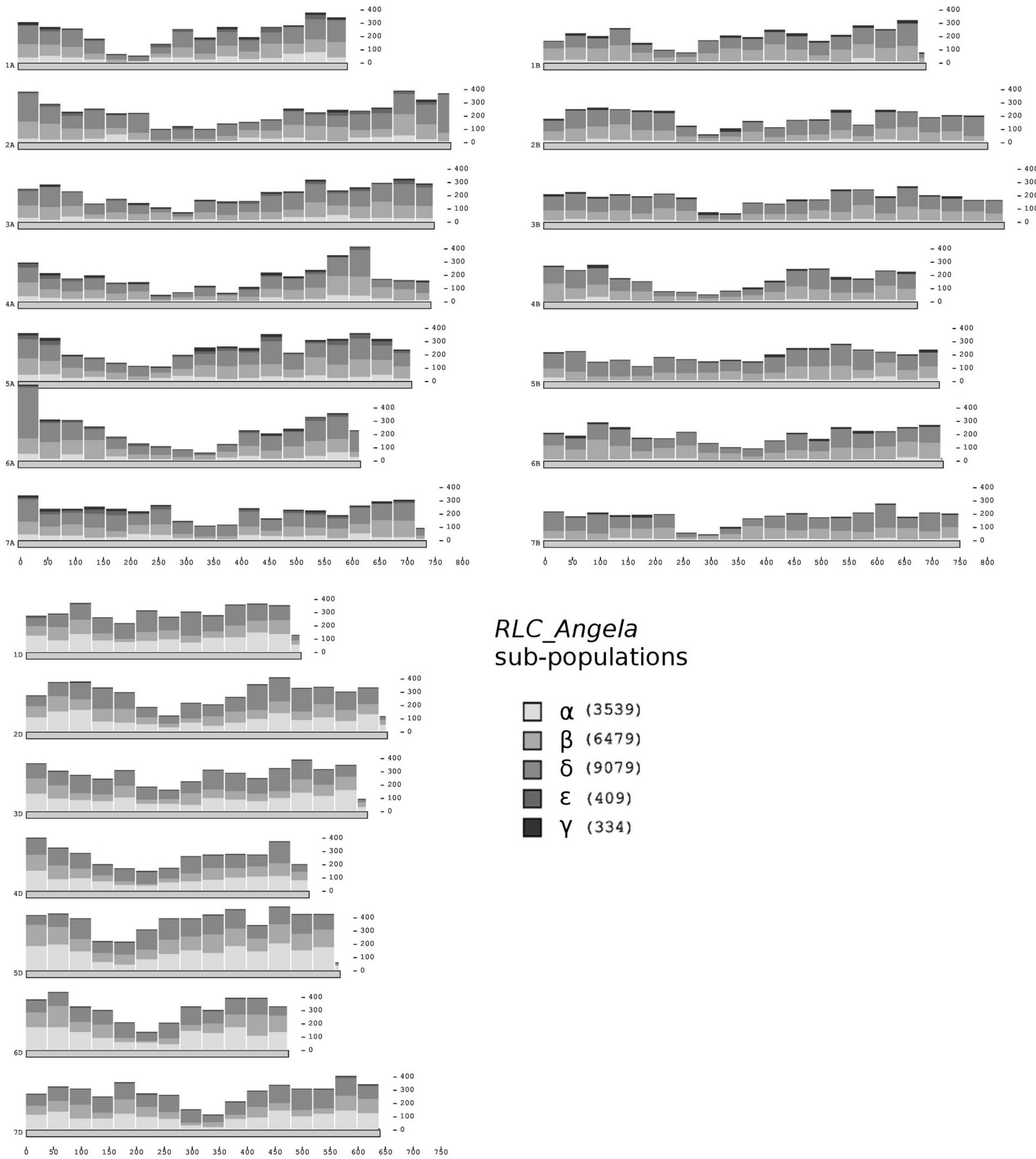

Supplementary Fig. 6. Distribution of *RLC_Angela* sub-populations along wheat chromosomes in bins of 40 Mb. Note that the abundance of elements from the *RLC_Angela* sub-populations differs in the three wheat sub-genomes. The x-axis indicates the position in Mb, the y-axis indicates the number of retrotransposon copies per bin.

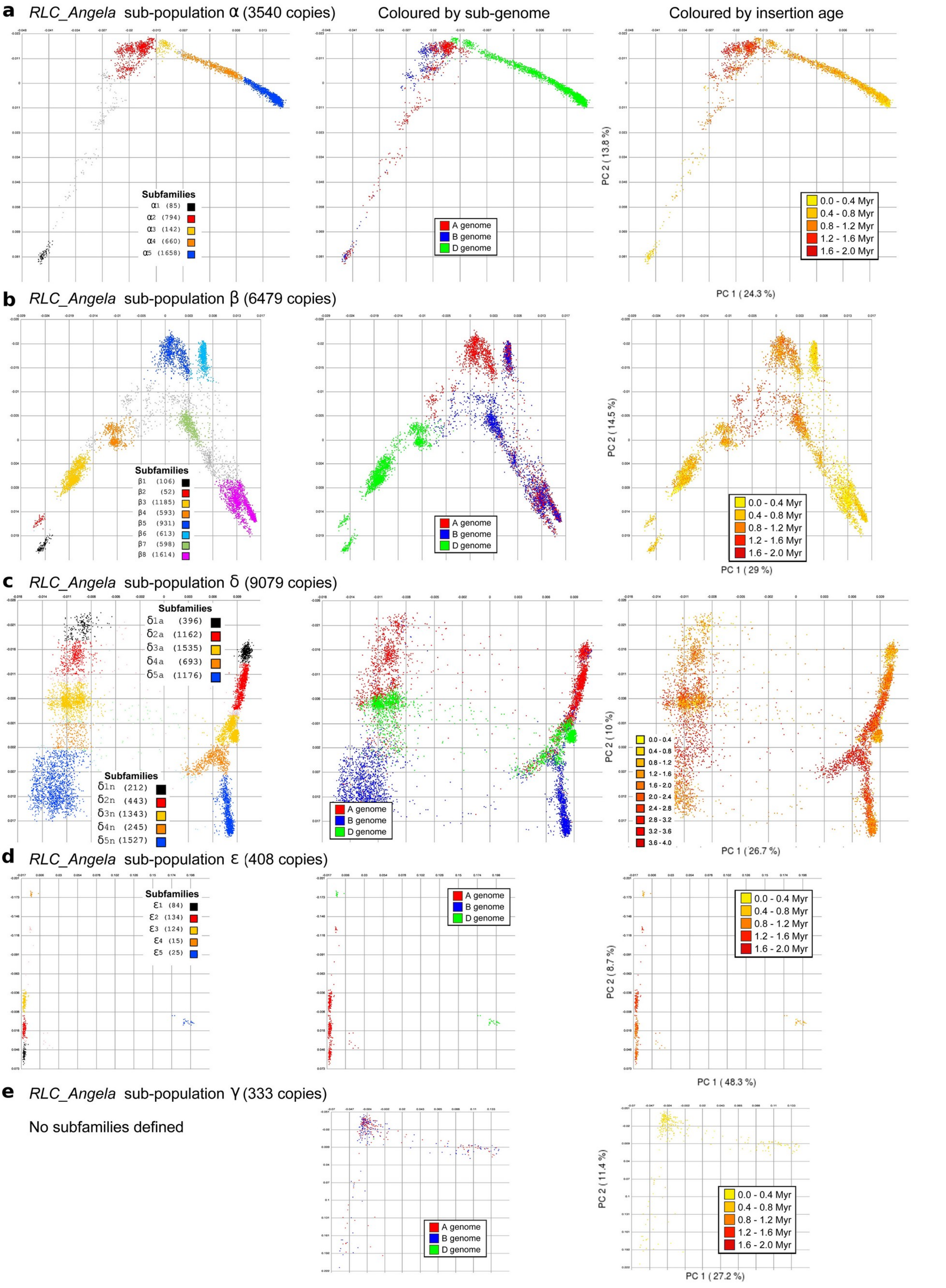

Supplementary Fig. 7. Definition of subfamilies through PCA in individual sub-populations of *RLC_Angela* retrotransposons (x-axis: PC1, y-axis: PC2, with percentage of variation explained by each PC indicated in the rightmost panel). Panels **a.** through **e.** show how sub-populations α, β, δ, ε and γ were classified into different subfamilies. Shown are sub-family definitions (left), subgenome localization (center) and insertion age (right) for all sub-populations.

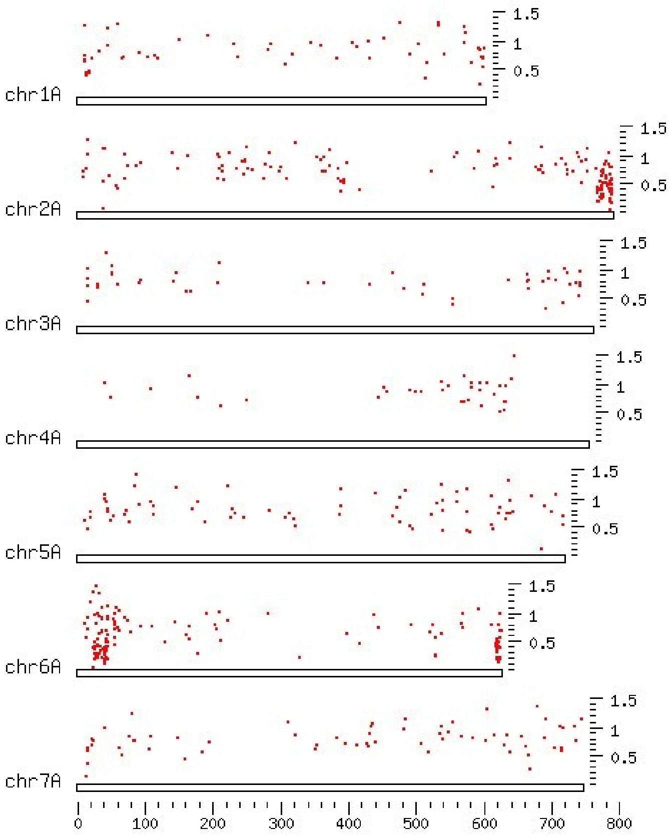

Supplementary Fig 8. Distribution of 604 *RLC_Angela* retrotransposons of the δ1a and δ1n subfamilies in the A genome of wheat. Individual retrotransposon copies are indicated with red dots. The x-axis indicates the physical position, while the y-axis indicates the insertion age in million years. Note that the left end of chromosomes 2A and both ends of chromosome 6A are highly enriched in δ1a and δ1n elements, indicating chromosomal introgressions from a different wheat relative. Additionally, these introgressed segments contain many more younger elements that the rest of the genome, indicating that they were active in more recent times in the donor species. This also indicates that δ1a and δ1n elements have not spread much across the wheat genome after the introgression events.

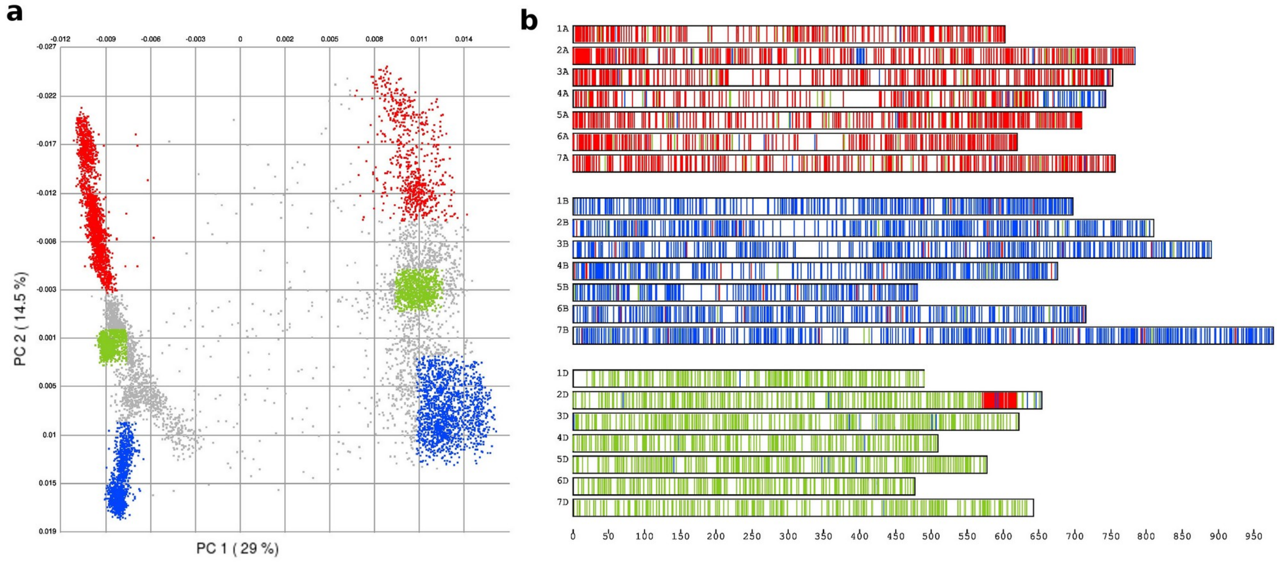

Supplementary Fig. 9. Distribution of *RLC_Angela* sub-families in wheat sub-genomes of wheat line Arina*LrFor*. Here, only elements of *RLC_Angela* sub-population δ are shown. **a.** PCA of *RLC_Angela* sub-population δ elements. PC 1 at the horizontal axis separates autonomous and non-autonomous elements, while PC 2 largely separates elements from the wheat sub-genomes. The percentage of variation explained by each PC is given in parentheses. **b.** Mapping of the *RLC_Angela* sub-families on to wheat chromosomes. The x-axis indicates the position in Mb. Note that translocations from the B to the A genome are visible on chromosomes 2A and 4A. These are common to all 10 sequenced wheat lines. Additionally, an introgression of a segment coming from an A genome relative is visible on chromosome 2D.

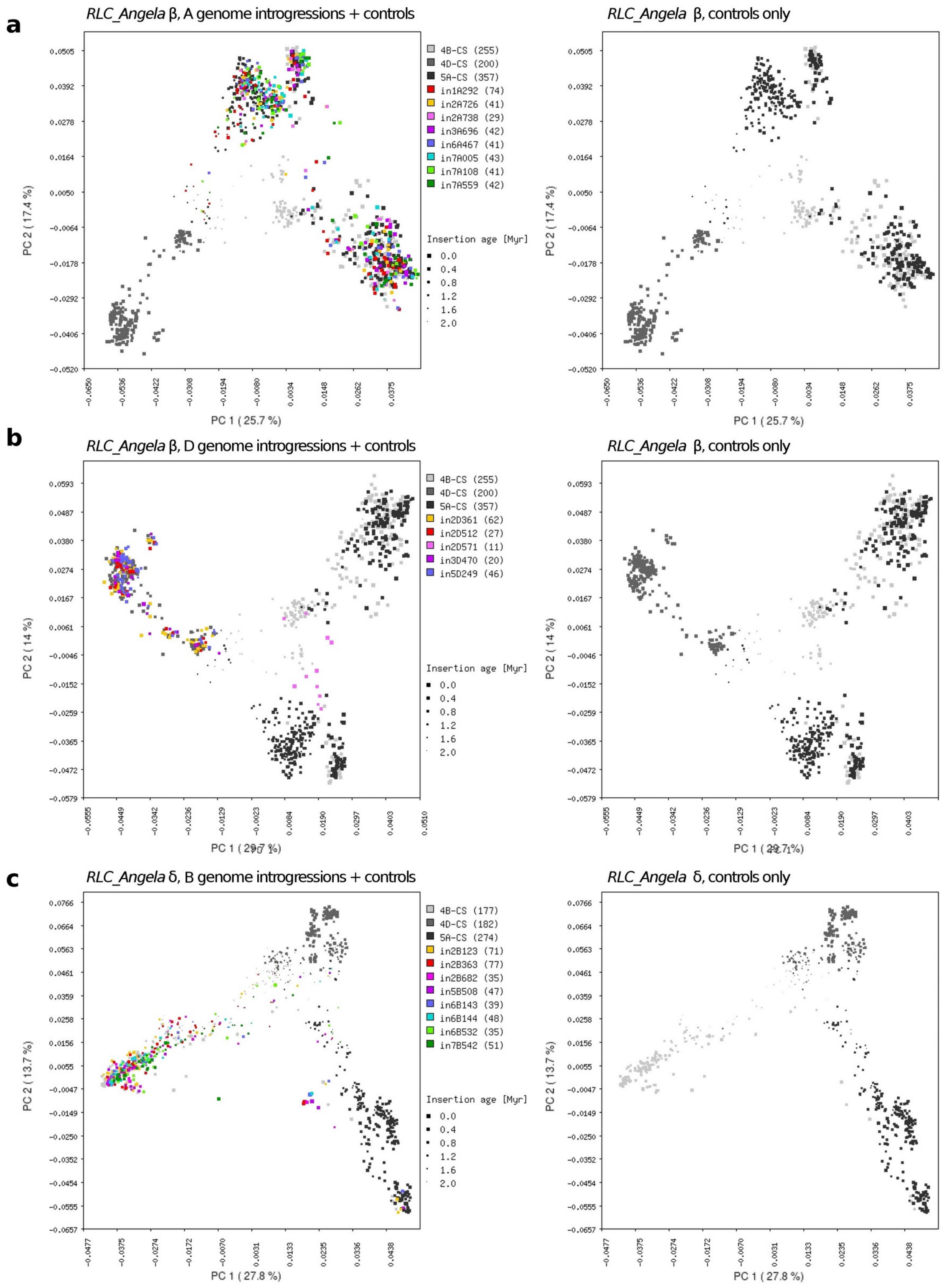

Supplementary Fig. 10. Characterization of chromosomal introgressions in wheat using PCAs of *RLC_Angela* retrotransposon populations. In each left panel, retrotransposons from identified introgression segments are shown as colored dots with younger elements being indicated as larger dots. The percentage of variation explained by each PC is given in parentheses. In shades of gray are retrotransposons from the A, B and D subgenomes from introgression-free segments of the wheat genome backbone, serving as controls. The right hand panels show only the A, B and D genome controls to make it easier to identify retrotransposons that were introgressed from different genetic backgrounds see populations of retrotransposons that form. Introgressions names have the prefix "in" followed by the chromosome name and the start point of the introgression (in Mb). **a.** *RLC_Angela* sub-population β elements in A genome introgressions. **b.** *RLC_Angela* sub-population β elements in D genome introgressions. **c.** *RLC_Angela* sub-population δ elements in B genome introgressions.

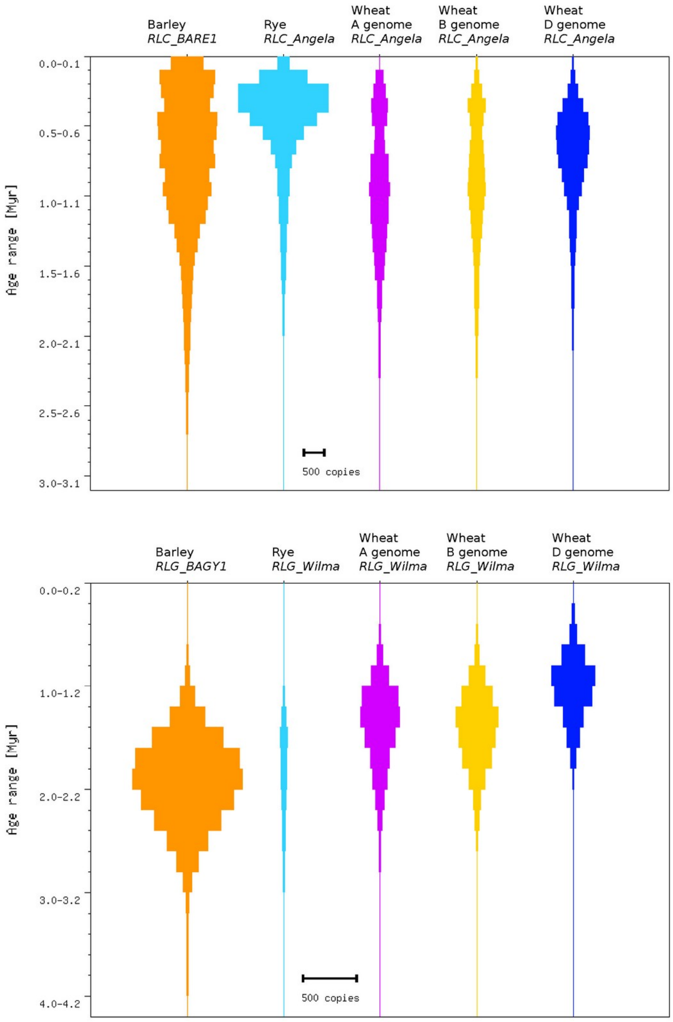

Supplementary Fig. 11. Comparison of activity times of *RLC_Angela* (top) and *RLG_Wilma* retrotransposons from wheat sub-genomes and their homologs from wheat and barely. Population sizes of retrotransposon families over time are shown by violin plots. Note that the TE families were active at different times and to different degrees in the three species and even in the three wheat sub-genomes.

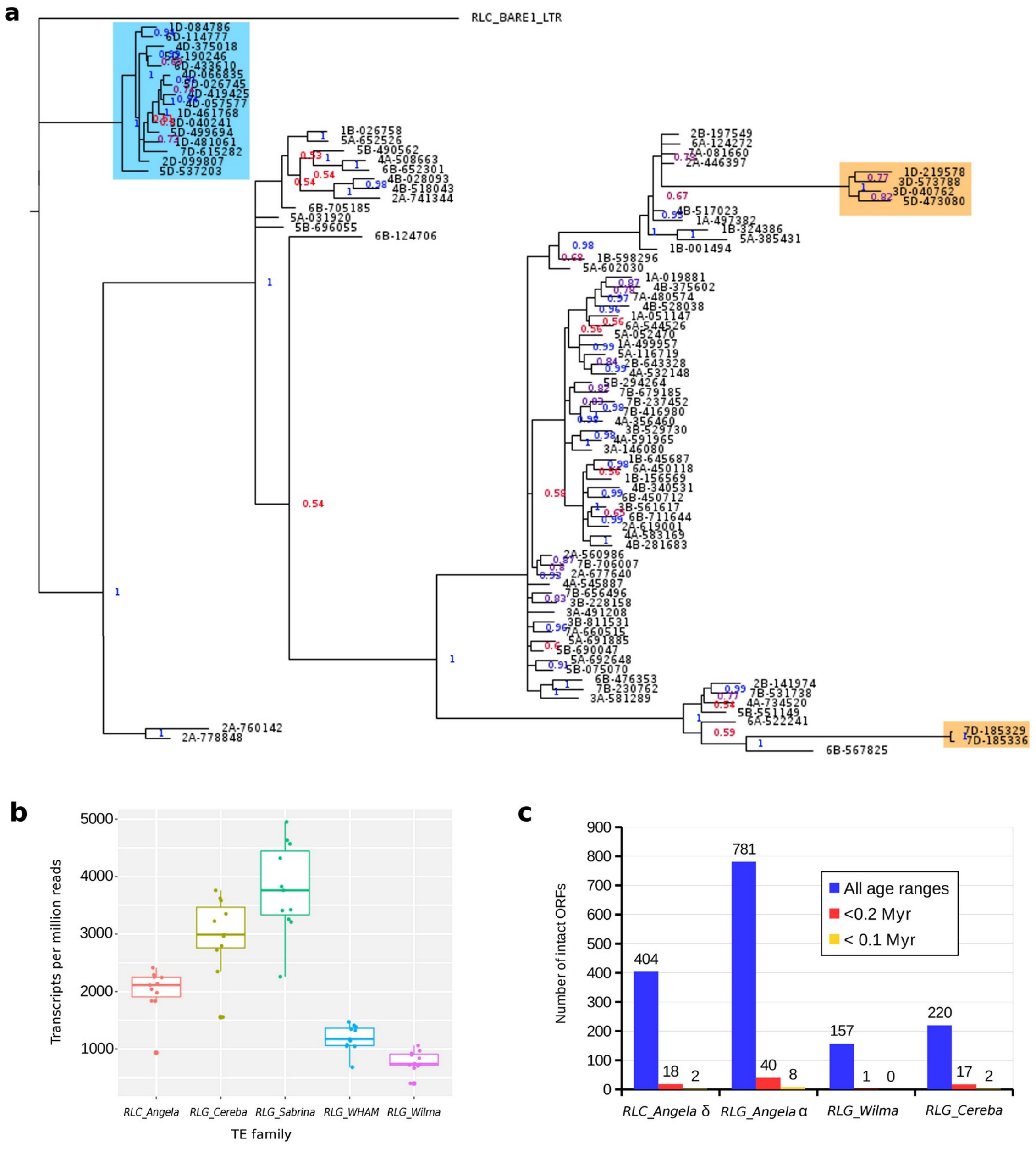

Supplementary Figure 12. Evidence for recent retrotransposon acitivity. **a.** Phylogenetic tree of 99 *RLC_Angela* sub-population β elements which are younger than 100,000 years. Retrotransposon copies that were present in the D-genome form a basal clade (highlighted in blue). A and B-genome retrotransposon which invaded the D-genome after hexaploidization form distinct clades (highlighted in orange). b. Expression levels of retrotransposon families in four transcriptome experiments (3 replicates each). Each dot represents one transcriptome sample. The transcriptome data was derived from wheat infected with 3 different isolates of powdery mildew as well as control plants. Since there were no significant differences between any of the condition and samples, they are not especially labeled. **c.** Numbers of intact open reading frames (ORFs) in *RLC_Angela* (α and sub-populations), *RLG_Wilma* and *RLG_Cereba* retrotransposons.
